# Smartphone-Based 3D Surface Imaging: A New Frontier in Digital Breast Assessment? Smartphone-Based Breast Assessment

**DOI:** 10.3390/jcm14176233

**Published:** 2025-09-03

**Authors:** Nikolas Chrobot, Philipp Unbehaun, Konstantin Frank, Michael Alfertshofer, Wenko Smolka, Tobias Ettl, Alexandra Anker, Lukas Prantl, Vanessa Brébant, Robin Hartmann

**Affiliations:** 1 Center of Plastic, Aesthetic, Hand and Reconstructive Surgery, University Hospital Regensburg, Franz-Josef-Strauß-Allee 11, 93053 Regensburg, Germanypunbehaun@csj.de (P.U.); lukas.prantl@ukr.de (L.P.); 2 Department of Plastic Surgery and Hand Surgery, Technical University Munich, 81675 Munich, Germany; 3 Department of Oral and Maxillofacial Surgery and Facial Plastic Surgery, Ludwig-Maximilians-University (LMU), Lindwurmstrasse 2a, 80337 Munich, Germany; 4 Department of Oral and Maxillofacial Surgery, University Hospital Regensburg, Franz-Josef-Strauß-Allee 11, 93053 Regensburg, Germany

**Keywords:** three-dimensional surface imaging, stereophotogrammetry, smartphone-based surface imaging, digital anthropometry, plastic surgery, Vectra H2

## Abstract

**Background**: Three-dimensional surface imaging is widely used in breast surgery. Recently, smartphone-based approaches have emerged. This investigation examines whether smartphone-based three-dimensional surface imaging provides clinically acceptable data in terms of accuracy when compared to a validated reference tool. **Methods**: Three-dimensional surface models were generated for 40 patients who underwent breast reconstruction surgery using the Vectra H2 (Canfield Scientific, Fairfield, NJ, USA) and the LiDAR sensor of an iPhone 15 Pro in conjunction with photogrammetry. The generated surface models were superimposed using CloudCompare’s ICP algorithm, followed by 14 linear surface-to-surface measurements to assess agreement between the three-dimensional surface models. Statistical methods included absolute error calculation, paired *t*-test, Bland–Altman analysis, and Intra-Class Correlation Coefficients to evaluate intra- and inter-rater reliability. **Results**: The average landmark-to-landmark deviation between smartphone-based and Vectra-based surface models was M = 2.997 mm (SD = 1.897 mm). No statistical differences were found in 13 of the 14 measurements for intra-rater comparison and in 12 of the 14 for inter-rater comparison. The Intra-Class Correlation Coefficient for intra-rater reliability of the iPhone was good, ranging from 0.873 to 0.993. Intra-Class Correlation Coefficient values indicated good reliability, ranging from 0.873 to 0.993 (intra-rater) and 0.845 to 0.992 (inter-rater). Bland–Altman analyses confirmed moderate to reliable agreement in 13 of 14 measurements. **Conclusions**: Smartphone-based three-dimensional surface imaging presents promising possibilities for breast assessment. However, it may not yet be suitable for highly detailed breast assessments requiring accuracy below the 3 mm threshold.

## 1. Introduction

Surgical procedures involving the breast require a profound understanding of subtle changes in volume and morphology. Consequently, three-dimensional (3D) surface imaging has become a fundamental tool in breast assessment in the context of plastic surgery [1,2,3,4]. Despite MRI being considered the absolute gold standard, 3D surface imaging offers a reproducible and strong correlation to MRI volumes [2,5]. Regardless of the imaging method employed, these techniques provide detailed insights into breast morphology and symmetry and are utilized in a wide range of clinical scenarios [1,6,7].

The advent of smartphone-based 3D surface imaging allows for easy and cost-effective acquisition of surface models (SMs). However, limited research exists validating this technique for clinical use in breast reconstruction surgery (BRS). Rudy et al. conducted a study utilizing the iPhone X (Apple Inc., Cupertino, CA, USA) and the Vectra H2 (Canfield Scientific, USA) to compare anthropometric measurements between nine anatomical landmarks in ten patients undergoing BRS [7]. The measurements revealed good agreement, with an overall average discrepancy of 0.91 mm ± 0.073 mm between the two methods. They concluded that the smartphone was capable of capturing breast measurements with an average discrepancy of less than 2.0 mm and emphasized the need for further research, particularly in the development of suitable software.

Han et al. developed specialized software for iOS (Apple Inc., USA) and used an iPhone 12 Pro to acquire SMs from 46 patients [8]. They conducted nine direct manual measurements of the breast and compared them to those performed on the smartphone-based SMs. The authors found a relative technical error of measurement (rTEM) ranging from 2.99% to 5.19% for most measurements, while nipple-to-inframammary fold (IMF) distances showed poor rTEM, reaching up to 19.76%. They concluded that their iOS-based software, utilizing the iPhone’s LiDAR sensors, provided an ideal 3D scanning solution that was affordable, accurate, portable, and easy to use. Despite previous pioneering research providing a comprehensive overview, detailed surface-to-surface distance assessments were not sufficiently described in earlier investigations.

This study compares smartphone-based 3D SMs obtained using an iPhone 15 Pro with an evaluated and established tool in portable 3D surface imaging, the Vectra H2. Surface-to-surface distances were acquired by superimposing the 3D SMs to assess agreement between the two methods.

The comparison between the SMs generated by the two methods involved thorough calculations of surface-to-surface distances.

## 2. Material and Methods

### 2.1. Study Protocol

This prospective, monocentric study was conducted according to the guidelines of the Declaration of Helsinki at the Department of Plastic, Aesthetic, Hand, and Reconstructive Surgery at Hospital St. Josef Regensburg, Germany, after obtaining approval from the ethics committee of the University of Regensburg (reference number: 20-1653_3-101; date of approval: 2 January 2024). This study was registered in the German Clinical Trials Register (DRKS) under the registration number DRKS00034221 on the 10 May 2024. A total of 40 patients who underwent BRS at the institution between 1 October 2018 and 1 October 2023 were enrolled in the study, as the aesthetic outcome is typically evaluated during this period. Patients with failed reconstructions or adhesive allergies were excluded from participation. Of the 43 patients screened for eligibility, three were excluded, two due to failed reconstruction and one due to adhesive allergies, resulting in a final sample of 40. While data acquisition and protocol were identical to a prior investigation, the present research evaluates deviations in surfaces by examining surface-to-surface distances, with novel processing and analysis of the data [9].

### 2.2. Participant Preparation

Eligible patients were invited to undergo a digital anthropometric examination, which included an explanation of the procedure and standardized positioning under controlled lighting conditions. To facilitate post-processing, 14 anatomical landmarks were marked with colored stickers, supplemented by four additional black stickers to support precise cropping after imaging.

**Figure 1:**
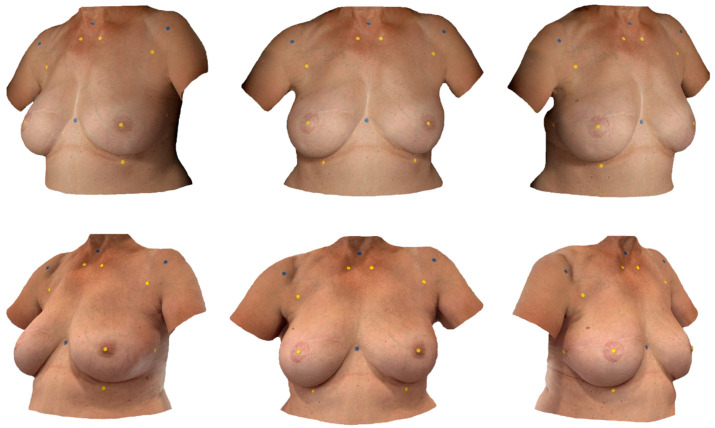
Surface Models: Appearance of a 58-year-old female patient after BRS on the right side. The figure compares the quality of the two different surface imaging systems from three different angles. The SM generated by the Vectra H2 is shown in the upper row, while the iPhone 15 Pro (Apple Inc., USA) in combination with the “3D-Scanner App” V2.1.2 (Laan Consulting Corp., Brooklyn, NY, USA) was used to create the SM in the lower row.

Figure 1 provides an overview of all landmarks and compares Vectra-based and smartphone-based SMs.

### 2.3. Three-Dimensional Data Acquisition

Three-dimensional SMs were obtained using the Vectra H2 and the iPhone 15 Pro in conjunction with the software 3D Scanner App V2.12 (Laan Consulting Corp., USA).

To generate the 3D SMs, the 3D Scanner App’s photo mode was employed, which combines photogrammetry with Apple’s LiDAR sensor. LiDAR uses time-of-flight measurements to determine the distance (or depth) between an object and the sensor. The Vectra H2 is a portable stereophotogrammetry system frequently used for breast assessment in plastic surgery [10]. The system has been validated in previous investigations, demonstrating sufficient accuracy for breast assessment [11,12]. In accordance with a previously described protocol, patients were provided with a stick positioned horizontally, with their arms at a 45° angle to the ground, to minimize arm movement during the scans [9,13].

SMs were obtained sequentially by the same operator, who conducted all scans in a designated 3D scanning room designed for pre- and postoperative surgical consultations.

### 2.4. Three-Dimensional Data Processing

To process the data, both SMs were exported to CloudCompare (http://cloudcompare.org/ accessed on 14 April 2024) as Wavefront OBJ files. To ensure consistency, 3D SMs were cropped at predefined anatomical boundaries involving the throat, arms, and the upper abdominal area. After manually matching the 3D SMs, CloudCompare’s ICP implementation was conducted to ensure proper scaling. The 3D SMs were then exported into Meshlab (ISTI-CNR, Pisa, Italy) for further processing [14]. To conduct measurements, normals had to be computed for point sets, followed by the Screened Poisson algorithm. For superimposition and assessing surface-to-surface distances, the 3D SMs were imported into the VECTRA Analysis Module (VAM) software (https://www.canfieldsci.com/imaging-systems/vectra-h2-3d-imaging-system/, accessed on 17 July 2025) as Wavefront OBJ-files.

### 2.5. SM Comparison

The VAM software was utilized to evaluate absolute discrepancies between the superimposed SMs. SM comparison involved measuring the surface-to-surface distance between corresponding anatomical landmarks. Table 1 provides an overview of all performed measurements.

**Figure 2:**
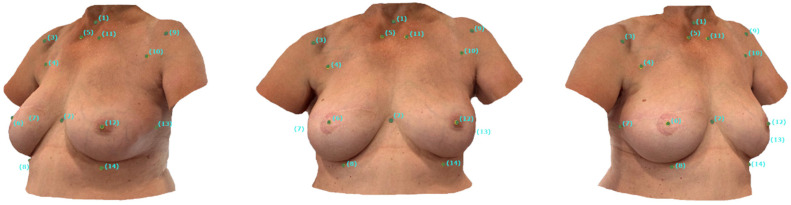
Digital Landmarks: Appearance of a 58-year-old female participant after DIEP flap BRS on the right. After transferring the smartphone-based SM into VAM, the landmarks were manually set in the center of the sticker to subsequently assess the landmark-to-landmark distance utilizing the VAM software. The landmarks are labeled as described in Table 1.

The anatomical landmarks were digitized in VAM by manually marking the center of the stickers on both SMs. The surface-to-surface analysis was performed by measuring the distance between the corresponding landmarks on the superimposed SMs. Figure 2 illustrates the digital landmarks set by a medical professional at the center of the sticker on a smartphone-based SM.

**Figure 3:**
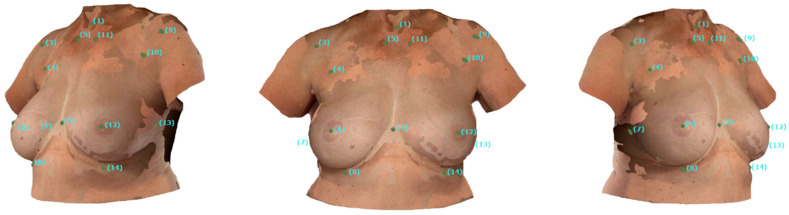
Presentation of the Superimposed SMs: The figure depicts the superimposed SMs of a 58-year-old female patient after BRS on the right. Superimposition was performed using Vectra’s VAM software. The manually placed digital landmarks show high alignment of the SMs. Nevertheless, landmarks (3) and (7) exhibit a heightened deviation, likely due to small movements between the scans. The landmarks are labeled as shown in Table 1.

Intra-rater reliability was assessed by repeating all surface-to-surface distance measurements by the same operator. To evaluate interrater reliability, the same procedure was performed by a different operator. Figure 3 depicts the superimposed SMs of the smartphone-based and Vectra-based SMs.

### 2.6. Statistical Analysis

Statistical analyses were conducted using IBM SPSS Statistics 27 (SPSS Inc., Chicago, IL, USA). The primary objective was to compare landmark-to-landmark distances between the smartphone-based and Vectra-based SMs to assess their accuracy. A threshold of 3 mm was defined as the clinically acceptable limit, in alignment with previous studies [15,16]. Deviations exceeding 3 mm were considered clinically significant. For each measurement, average landmark-to-landmark distances were calculated. The Intra-Class Correlation Coefficient (ICC) was used to assess intra- and inter-rater reliability. The ICC was classified according to Koo and Li as follows: less than 0.5 as poor, values between 0.5 and 0.75 as moderate, values between 0.75 and 0.9 as good, and values greater than 0.90 as excellent reliability [17]. To examine the normality of the data, the Kolmogorov–Smirnov test was utilized. Subsequently, a paired *t*-test was applied to evaluate the mean values of the data. Agreement between intra- and inter-rater measurements was further examined using Bland–Altman analysis. The 95% limits of agreement (LoA) were interpreted according to the classification by Aung et al.: mean differences exceeding 2 mm were deemed unreliable, differences between 1.6 mm and 2 mm were considered moderately reliable, those between 1 mm and 1.5 mm were regarded as reliable, and mean differences below 1 mm were considered highly reliable [18].

**Figure 4:**
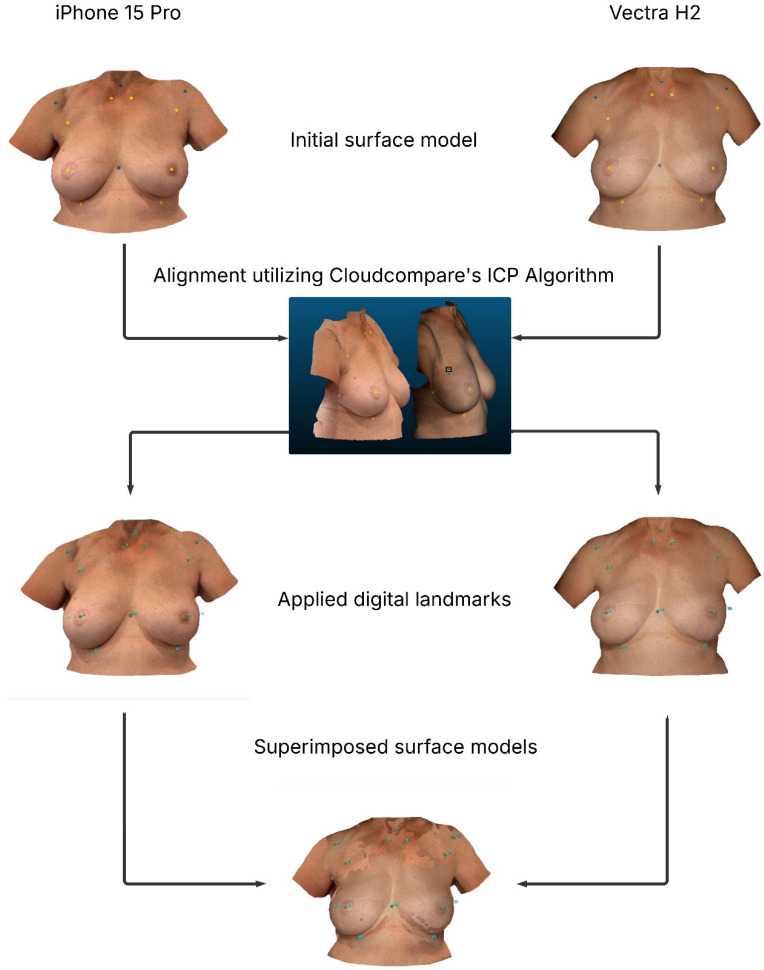
Flowchart: The figure describes the step-by-step process both SMs underwent to generate the superimposed SMs and to set up for assessing the measurements. The left column shows the SM generated by the iPhone 15 Pro, whereas the right column depicts the process of the Vectra-based SM.

Figure 4 illustrates the processing steps of the SMs as a flowchart.

## 3. Results

### 3.1. Patient Demographics

The cohort included 40 female participants. This study focused on postoperative breast reconstruction patients to evaluate the technology in its intended clinical setting, considering real-world factors like scarring and altered morphology. Their mean age was M = 51.5 years (SD = ±8), mean height M = 167 cm (SD = ±6 cm), mean weight M = 73.2 kg (SD = ±13.4 kg), and mean BMI M = 26.3 (SD = ±4.2). All patients underwent BRS with a deep inferior epigastric perforator flap reconstruction. Thirteen patients underwent immediate delayed, thirteen immediate, and 14 delayed BRS. Three patients underwent bilateral BRS, while 37 were reconstructed unilaterally. Five patients underwent skin-sparing mastectomy, three underwent nipple-sparing mastectomy, and 32 patients underwent modified radical mastectomy before BRS. The average flap weight was 622.6 g ± 146.4 g on the right side and 573 g ± 395.6 g on the left side.

### 3.2. Landmark-to-Landmark Distance Analyses

Table 2 presents the outcomes of the landmark-to-landmark distance analyses. The overall landmark-to-landmark distance between smartphone- and Vectra-based SMs was calculated at M = 2.997 mm (SD = 1.897 mm). Landmarks (1), (2), (4), (5), (6), (10), (11), (12), and (14) exhibited clinically acceptable landmark-to-landmark deviations of less than 3 mm. However, the CP (R) and (L) (3) and (9), the LaBP (R) and (L) (7) and (13), as well as the LBP (R), exhibited clinically unacceptable deviations of more than 3 mm. The maximum landmark-to-landmark distance was found in landmark (9), the CP (L), with an average landmark-to-landmark deviation of M = 5.20 mm (SD = 3.56 mm).

### 3.3. Intra- and Inter-Rater Reliability

Table 3 presents the descriptive statistics for the intra-rater reliability of landmark-to-landmark distance analyses. The overall landmark-to-landmark distance between the two SMs was M = 2.96 mm (SD = 1.96 mm). Landmarks (1), (2), (4), (5), (6), (7), (10), (11), (12), and (14) were below the 3 mm threshold, while measurements (3), (8), (9), and (13) exceeded 3 mm (Table 3). The ICC for the iPhone’s intra-rater reliability was good, ranging from 0.873 to 0.993 (Table 4). The Kolmogorov–Smirnov test indicated normal distribution for all measurements except for measurement (9). Due to the absence of normality, the Wilcoxon signed-rank test was employed to calculate central tendencies for the intra-rater data. The *t*-test for paired samples revealed no significant differences in mean values for all measurements except for measurement (9) (Table 5). Given the non-normal distribution of measurement (9), the Wilcoxon signed-rank test was performed: (9): (M = 5.20 vs. 4.92; *p* = 0.002). Bland–Altman analysis of the intra-rater data yielded promising results, with only measurement (4) classified as unreliable according to Aung et al. [18]. Twelve of the 14 measurements were deemed reliable, with 95% Bland–Altman LoA ranging between 1 mm and 1.5 mm. Measurement (10) was classified as moderately reliable, with 95% Bland–Altman LoA between 1.6 mm and 2 mm (Table 4).

Table 6 displays the outcomes of the landmark-to-landmark analyses conducted by the inter-rater. The overall landmark-to-landmark distance was calculated at M = 3.07 mm (SD = 1.99 mm), which is consistent with previous findings. The inter-rater identified mean distances below 3 mm in eight landmarks (1), (2), (4), (5), (6), (10), (11), and (12), while the remaining six landmarks (3), (7), (8), (9), (13), and (14) exhibited mean distances > 3 mm. However, the ICC values for inter-rater reliability were excellent, ranging from 0.845 to 0.992 (Table 4). The Kolmogorov–Smirnov test indicated normal distribution for all measurements except for measurement (4). The Wilcoxon signed-rank test was conducted to assess central tendencies for the inter-rater comparison. The *t*-test for paired samples showed no significant differences in mean values for any of the measurements, except for measurement (4). Given the non-normal distribution of measurement (4), the Wilcoxon signed-rank test revealed a significant difference (M = 2.26 vs. 2.54; *p* = 0.035) (Table 7).

Table 4 presents the Bland–Altman analyses conducted using the inter-rater data. The 95% Bland–Altman LoA for the inter-rater showed similar trends to those observed in the intra-rater analysis, with the highest disparity noted in measurement (4) (1.95 mm and −2.53 mm) and the lowest disparity in measurements (2) (0.94 mm and −1.06 mm) and (3) (1.07 mm and −0.93 mm). According to Aung et al., ten of the 14 measurements were considered reliable, two were classified as moderately reliable, and only measurement (4) was deemed unreliable [18].

## 4. Discussion

This study aimed to evaluate whether smartphone-based 3D surface imaging provides clinically acceptable accuracy for breast assessment compared to the portable stereophotogrammetry system Vectra H2. To evaluate accuracy, both SMs were superimposed, and surface-to-surface distances were assessed. Overall, the landmark-to-landmark deviation was 2.997 mm on average, with the majority of measurements within the predefined 3 mm threshold. Despite the promising results, this trial warrants further discussion. Although the patient cohort overlaps entirely with a previous study, the current research employs a fundamentally different methodological approach by utilizing surface-to-surface deviation analysis rather than traditional linear anthropometric measurements [9]. This approach allows for a different type of morphological assessment and provides complementary insights beyond those addressed in the prior analysis. Despite MRI being considered as the gold standard, the Vectra H2 was utilized as the reference method because, despite its lower accuracy, the clinical application of smartphone-based 3D surface imaging is more comparable to that of the Vectra H2, especially in terms of cost and accessibility [2]. Limited data exist on surface-to-surface distances of SMs of the breast. However, comparisons to similar studies assessing facial accuracy are feasible. Seifert et al. investigated the accuracy of various applications for the iPhone 14 Pro, comparing the generated SMs to a stationary system considered the gold standard, the 3dMD (3dMD Research Limited, Wexford, Ireland) [19]. They reported that the mean surface distance of smartphone-based 3D SMs ranged between 1.46 mm and 1.66 mm, depending on the software used. The smaller deviations observed in their study could be attributed to changes in patient positioning, which can introduce discrepancies unrelated to the scanning method itself.

De Stefani et al. noted that small movements during facial scans can affect the accuracy of the generated models [20]. Although this effect has been discussed in previous investigations regarding facial expressions during or between 3D scans, the absolute deviation in surface-to-surface distances could be even greater during breast assessments [20,21]. There are multiple possible reasons for that. As discussed in a prior study, changes in shoulder position can cause shifts in soft tissue, particularly in the breast [9]. This effect is amplified in patients with larger breast volumes or ptosis. A similar study evaluating the reliability of smartphone-based volumetry in comparison with MRI reported comparable findings, with discrepancies increasing in patients with larger breast volumes [22]. The resulting error, together with deviations in shoulder position, is transferred to the scaling process, thereby affecting accuracy during superimposition. This effect is particularly evident in measurements (3) and (9), where the highest mean errors were detected, followed by the LaBP (7) and (13) on both sides due to shifts in breast position.

Additionally, greater variability in 3D SMs can arise from motion artifacts, especially affecting the thorax due to natural respiration. In comparison to the Vectra H2, smartphone-based surface imaging may even excel in managing respiratory movements. While the Vectra H2 generates 3D SMs based on three acquired images, the iPhone 15 Pro captures a series of images during the scan. This approach smooths the generated 3D SMs and may mitigate the impact of respiratory motion, thereby enhancing the comparability of postoperative surface assessments. Furthermore, despite the surface imaging being conducted in the same clinical setting, minor variations in lighting could have led to slight inaccuracies. Seifert et al. also demonstrated that the software used on the iPhone affects the accuracy of the generated 3D SMs [19]. Consequently, while central tendencies found in current investigations remain consistent across different devices and applications, 3D surface imaging software, particularly those developed for clinical applications, must be evaluated independently.

A previous study conducted at our institution involving 30 healthy individuals identified discrepancies between digital anthropometric measurements of the face and those acquired using the iPhone 14 Pro [23]. Consequently, 15 landmark-to-landmark distances were assessed between the superimposed SMs. Photogrammetry-based SMs showed an average discrepancy of M = 0.8 mm, with 95% Bland–Altman LoA consistently below 2 mm. This indicates that surface-to-surface distances appear smaller when comparing facial 3D SMs. Additionally, the 95% Bland–Altman LoA yielded similar results. In conclusion, despite the larger deviations in mean values, the reliability appears comparable. This may be attributed to the larger surface area of the breast SMs, which could lead to greater absolute errors during superimposition. Consequently, minor local misalignments may accumulate as the area increases, resulting in higher overall deviations.

Thurzo et al. reported several deviations exceeding 3 mm in SMs acquired from 60 patients when comparing the Bellus 3D Dental Pro application, using the iPhone 12 Pro, to SMs generated by cone beam computed tomography [15]. Based on these findings, they concluded that smartphone-based 3D surface imaging has limited clinical relevance. However, it may still be useful in clinical applications where sub-3 mm accuracy is not required, whereas in aesthetic applications, deviations ≥ 3 mm may already be clinically relevant. For instance, Rudy et al. reported an average RMS deviation of 1.85 mm and a mean absolute measurement discrepancy of 0.91 mm ± 0.073 mm [7]. However, these findings were based on a sample of ten patients, suggesting that previous studies might have overestimated the accuracy of smartphone-based surface imaging. Although flap weights were recorded, they were not incorporated into the validation process because postoperative factors such as swelling, edema, tissue remodeling, particularly given the varying time intervals since surgery among patients, can alter volume and limit the accuracy of direct comparisons to intraoperative weights. Previous work has also demonstrated the feasibility of estimating breast volume from anthropometric measurements, as in the BREAST-V formula, which was developed and validated against mastectomy specimen weights [24]. Future research should also investigate the accuracy of smartphone-based 3D surface imaging for volumetric assessment.

Furthermore, inconsistencies in the definition of clinical acceptability are reported throughout the literature. Aung et al. proposed that deviations greater than 2 mm are clinically unreliable, based on comparisons between direct anthropometric measurements and those obtained using an optical surface scanner developed at University College Hospital, London [18]. Thurzo et al. considered differences exceeding 3 mm to be unacceptable in their evaluation of TrueDepth-based 3D surface imaging [15]. While 3D surface imaging is useful in many scenarios, it should not be relied on exclusively. Surgically relevant information, such as skin texture, cannot be sufficiently assessed using 3D surface imaging. Therefore, 3D SMs should be considered as a complementary tool in some clinical applications, such as preoperative planning. Although both approaches require a similar amount of time to capture and generate the 3D surface models, with approximately 1 min, the preprocessing of smartphone-derived scans can be labor-intensive. On average, this step takes around 30 min, which currently limits clinical application due to the unjustified additional workload until specialized software is developed. Such specialized software should also ensure precise metric scaling, as many commercially available smartphone apps prioritize rendering quality over measurement accuracy. Nonetheless, independent validation remains essential for any newly developed applications prior to clinical implementation. For this study, due to the lack of reference data and the use of a relatively new method with significant potential for further refinement, the 3 mm threshold was applied when assessing surface-to-surface deviations, as it provides a pragmatic benchmark aligned with clinical relevance observed in related anthropometric applications [15]. Moreover, this technology may be employed in clinical settings where absolute precision below 2 mm is not necessary, such as routine pre- and postoperative documentation. Specifically, this technology could be implemented in clinics that have thus far lacked access to significantly more expensive reference systems. In this field of application, this more affordable method could still gain acceptance despite its currently lower precision, as the SMs provide sufficient information to enable improved planning or follow-up.

## 5. Conclusions

This study demonstrates that smartphone-based 3D surface imaging, while currently less precise than established systems such as the Vectra H2, offers promising potential for clinical application. The observed surface-to-surface deviations, although exceeding the 3 mm threshold in some regions, remain within a clinically acceptable range for the majority of anatomical points. Despite these limitations, the implementation of smartphone-based surface imaging may still enhance documentation or even surgical planning in clinical workflows. Moreover, the accessibility, affordability, and ease of use support its potential integration into clinical practice, especially for institutions operating with limited resources. This study contributes to clarifying the accuracy of smartphone-based surface imaging and its potential for clinical implementation.

## Figures and Tables

**Table 1:** Performed Measurements: The table presents the performed measurements in sequential order. Each row indicates the term used to describe the respective measurement and the anatomical localization of the associated landmark. All measurements were conducted on superimposed SMs based on the Vectra H2 and the iPhone 15 Pro. (R) = right side, (L) = left side.

Landmark-to-Landmark Distance	
Term	Landmark
(1) SN-SN	Sternal notch
(2) Xi-Xi	Xiphoid
(3) CP-CP (R)	Proc. Coracoideus (R)
(4) ULBP-ULBP (R)	Upper lateral breast point (R)
(5) UMBP-UMBP (R)	Upper medial breast point (R)
(6) Ni-Ni (R)	Nipple (R)
(7) LaBP-LaBP (R)	Lateral Breast point (R)
(8) LBP-LBP (R)	Lower Breast point (R)
(9) CP-CP (L)	Proc. Coracoideus (L)
(10) ULBP-ULBP (L)	Upper lateral breast point (L)
(11) UMBP-UMBP (L)	Upper medial breast point (L)
(12) Ni-Ni (L)	Nipple (L)
(13) LaBP-LaBP (L)	Lateral Breast point (L)
(14) LBP-LBP (L)	Lower Breast point (L)

**Table 2:** Descriptive Statistics: Landmark-to-landmark distance analyses; comparison of Vectra H2- and smartphone-based SMs. Values in mm. IBM SPSS 27 was used for data analysis.

Descriptive Statistics:					
	N	Minimum	Maximum	Mean	Std. Deviation
(1) SN-SN	40	0.05	6.99	2.73	1.79
(2) Xi-Xi	40	0.25	7.76	2.28	1.81
(3) CP-CP R	40	0.39	15.25	4.28	3.18
(4) ULBP-ULBP R	40	0.18	5.76	2.26	1.43
(5) UMBP-UMBP R	40	0.24	6.02	2.12	1.51
(6) Ni-Ni R	40	0.11	5.89	2.54	1.23
(7) LaBP-LaBP R	40	0.40	13.49	3.14	2.42
(8) LBP-LBP R	40	0.65	9.81	3.05	1.93
(9) CP-CP L	40	0.90	17.90	5.20	3.56
(10) ULBP-ULBP L	40	0.37	8.84	2.78	1.68
(11) UMBP-UMBP L	40	0.42	6.10	2.19	1.45
(12) Ni-Ni L	40	0.46	5.77	2.40	1.31
(13) LaBP-LaBP L	40	1.40	9.29	4.00	1.75
(14) LBP-LBP L	40	0.47	8.96	2.99	1.84
Overall	40	0.05	17.9	2.99	1.90

**Table 3:** Descriptive Statistics (Intra-rater): Landmark-to-landmark distance analyses of the data derived from the intra-rater. When compared to the original data, the intra-rater shows great consistency with an overall error in mean value of 0.03 mm; values in mm. IBM SPSS 27 was used for data analysis.

Descriptive Statistics (Intra-Rater)					
Intra-Rater	N	Minimum	Maximum	Mean	Std. Deviation
(1) SN-SN	40	0.29	8.17	2.71	1.95
(2) Xi-Xi	40	0.31	7.80	2.21	1.76
(3) CP-CP R	40	0.36	15.53	4.18	3.16
(4) ULBP-ULBP R	40	0.24	6.38	2.24	1.56
(5) UMBP-UMBP R	40	0.30	6.06	2.13	1.54
(6) Ni-Ni R	40	0.65	6.03	2.62	1.25
(7) LaBP-LaBP R	40	0.50	12.95	2.99	2.33
(8) LBP-LBP R	40	0.24	10.75	3.05	2.03
(9) CP-CP L	40	0.64	17.11	4.92	3.39
(10) ULBP-ULBP L	40	0.23	8.79	2.58	1.72
(11) UMBP-UMBP L	40	0.47	6.04	2.20	1.54
(12) Ni-Ni L	40	0.55	7.59	2.41	1.55
(13) LaBP-LaBP L	40	0.71	9.60	4.01	1.79
(14) LBP-LBP L	40	0.41	8.56	2.97	1.89
Overall	40	0.23	17.11	2.96	1.96

**Table 4:** Bland–Altman Analyses and ICC: 95% Bland–Altman LoA for measurements (1)–(14); values in mm. ICC values of the intra-rater and the inter-rater data compared to the values of the original measurements. IBM SPSS 27 was used for data analysis.

		Bland–Altman Analyses and ICC							
		ICC and Bland–Altman (Intra-Rater)				ICC and Bland–Altman (Inter-Rater)			
Variables				95% Confidence Interval				95% Confidence Interval	
	N	ICC (Intra-Rater)	Mean Bias	Upper Bound	Lower Bound	ICC (Inter-Rater)	Mean Bias	Upper Bound	Lower Bound
(1) SN-SN	40	0.973	0.014	1.21	−1.19	0.980	0.026	1.04 1.21	−0.99
(2) Xi-Xi	40	0.982	0.075	1.01	−0.86	0.980	−0.063	0.94 1.01	−1.06
(3) CP-CP R	40	0.994	0.097	1.06	−0.86	0.994	0.070	1.07 1.06	−0.93
(4) ULBP-ULBP R	40	0.873	−0.013	1.98	−2.01	0.845	−0.288	1.95 1.98	−2.53 −2.53 −2.53
(5) UMBP-UMBP R	40	0.970	−0.015	1.02	−1.05	0.971	−0.033	1.00	−1.06
(6) Ni-Ni R	40	0.960	−0.074	0.88	−1.02	0.940	−0.148	0.97	−1.26
(7) LaBP-LaBP R	40	0.979	0.156	1.49	−1.18	0.984	0.064 0.156	1.26 1.49	−1.13
(8) LBP-LBP R	40	0.970	0.001	1.33	−1.33	0.977	−0.069	1.09 1.33	−1.23
(9) CP-CP L	40	0.993	0.281 0.281	1.32	−0.76	0.992	0.189 0.281	1.34 1.32	−0.96
(10) ULBP-ULBP L	40	0.927	0.202	1.92	−1.52	0.924	−0.063	1.73 1.92	−1.85
(11) UMBP-UMBP L	40	0.964	−0.012	1.09	−1.11	0.964	−0.005 −0.012	1.12 1.09	−1.13
(12) Ni-Ni L	40	0.932	−0.012	1.42	−1.45	0.883	−0.053 −0.012	1.78 1.42	−1.89
(13) LaBP-LaBP L	40	0.978	−0.005	1.03	−1.04	0.971	−0.136 −0.005	1.04 1.03	−1.31
(14) LBP-LBP L	40	0.967	0.015	1.35	−1.32	0.953	−0.248 −0.498	1.27 1.35	−1.77

**Table 5:** T-Test for Paired Samples (Intra-rater): Comparison of Vectra H2-based and smartphone-based measurements between the original data and the intra-rater. * For measurement (9) Wilcoxon signed rank test was performed due to absence of normality of the data. IBM SPSS 27 was used for data analysis.

Variables		Paired Differences			
			95% Confidence Interval of the Difference		
	Mean	Std. Deviation	Lower	Upper	Two-Sided *p*
(1) SN-SN	0.0145	0.612	−0.181	0.210	0.882
(2) Xi-Xi	0.0753	0.475	−0.077	0.227	0.322
(3) CP-CP R	0.0966	0.490	−0.060	0.253	0.220
(4) ULBP-ULBP R	0.0129	1.017	−0.312	0.338	0.936
(5) UMBP-UMBP R	−0.0146	0.529	−0.184	0.154	0.863
(6) Ni-Ni R	−0.0739	0.484	−0.229	0.081	0.340
(7) LaBP-LaBP R	0.1558	0.679	−0.061	0.373	0.155
(8) LBP-LBP R	0.0009	0.680	−0.217	0.219	0.993
(9) CP-CP L	0.2813	0.529	0.112	0.450	0.002 *
(10) ULBP-ULBP L	0.2016	0.877	−0.079	0.482	0.154
(11) UMBP-UMBP L	−0.0119	0.563	−0.192	0.168	0.895
(12) Ni-Ni L	−0.0122	0.732	−0.246	0.222	0.917
(13) LaBP-LaBP L	−0.0046	0.530	−0.174	0.165	0.956
(14) LBP-LBP L	0.0147	0.680	−0.203	0.232	0.892

**Table 6:** Descriptive Statistics (Inter-rater): Landmark-to-landmark distance analyses of the data derived from the inter-rater. When compared to the original data, the inter-rater shows great consistency with an overall error in mean value of 0.08 mm; values in mm. IBM SPSS 27 was used for data analysis.

Descriptive Statistics (Inter-Rater)					
	N	Minimum	Maximum	Mean	Std. Deviation
(1) SN-SN	40	0.33	7.80	2.70	1.87
(2) Xi-Xi	40	0.10	8.73	2.34	1.81
(3) CP-CP R	40	0.97	15.08	4.21	3.19
(4) ULBP-ULBP R	40	0.05	7.72	2.54	1.72
(5) UMBP-UMBP R	40	0.26	6.65	2.15	1.60
(6) Ni-Ni R	40	0.51	6.39	2.69	1.21
(7) LaBP-LaBP R	40	0.30	12.96	3.08	2.41
(8) LBP-LBP R	40	0.34	10.49	3.12	1.99
(9) CP-CP L	40	0.94	16.77	5.01	3.49
(10) ULBP-ULBP L	40	0.59	9.67	2.85	1.73
(11) UMBP-UMBP L	40	0.38	6.02	2.20	1.57
(12) Ni-Ni L	40	0.47	6.93	2.45	1.55
(13) LaBP-LaBP L	40	1.15	10.80	4.14	1.87
(14) LBP-LBP L	40	0.18	8.01	3.24	1.92
Overall	40	0.10	16.77	3.07	1.99

**Table 7:** T-Test for Paired samples (Inter-rater): Comparison of Vectra H2-based and smartphone-based measurements between the original data and the inter-rater. * For measurement (4) Wilcoxon signed rank test was performed due to absence of normality of the data. IBM SPSS 27 was used for data analysis.

Variables		Paired Differences			
			95% Confidence Interval of the Difference		
	Mean	Std. Deviation	Lower	Upper	Two-Sided *p*
(1) SN-SN	0.026	0.518	−0.140	0.191	0.754
(2) Xi-Xi	−0.063	0.510	−0.226	0.100	0.436
(3) CP-CP R	0.070	0.508	−0.093	0.232	0.391
(4) ULBP-ULBP R	−0.288	1.142	−0.653	0.077	0.035 *
(5) UMBP-UMBP R	−0.033	0.525	−0.201	0.135	0.693
(6) Ni-Ni R	−0.148	0.570	−0.331	0.034	0.108
(7) LaBP-LaBP R	0.064	0.611	−0.131	0.259	0.511
(8) LBP-LBP R	−0.069	0.591	−0.258	0.120	0.464
(9) CP-CP L	0.189	0.589	0.001	0.378	0.049
(10) ULBP-ULBP L	−0.063	0.913	−0.355	0.229	0.667
(11) UMBP-UMBP L	−0.005	0.573	−0.188	0.179	0.959
(12) Ni-Ni L	−0.053	0.937	−0.352	0.247	0.723
(13) LaBP-LaBP L	−0.136	0.601	−0.328	0.056	0.160
(14) LBP-LBP L	−0.248	0.775	−0.496	−0.0002	0.05

## Data Availability

The data supporting the findings of this study consist of photographs of individuals. Due to privacy and ethical restrictions, these data cannot be made publicly available. Informed consent for the use of individual photographs was obtained from all participants.
